# Brain tumors and COVID-19: the patient and caregiver experience^*^

**DOI:** 10.1093/noajnl/vdaa104

**Published:** 2020-08-23

**Authors:** Mathew R Voisin, Kathy Oliver, Stuart Farrimond, Tess Chee, Jean Arzbaecher, Carol Kruchko, Mary Ellen Maher, Chris Tse, Rosemary Cashman, Maureen Daniels, Christine Mungoshi, Sharon Lamb, Anita Granero, Mary Lovely, Jenifer Baker, Sally Payne, Gelareh Zadeh

**Affiliations:** 1 Division of Neurosurgery, Department of Surgery, University of Toronto, Toronto, Ontario, Canada; 2 International Brain Tumour Alliance (IBTA), Tadworth, UK; 3 McMaster University, Hamilton, Ontario, Canada

**Keywords:** brain tumor, caregivers, COVID-19, patients, survey

## Abstract

**Background:**

Since the COVID-19 pandemic began, thousands of medical procedures and appointments have been canceled or delayed. The long-term effects of these drastic measures on brain tumor patients and caregivers are unknown. The purpose of this study is to better understand how COVID-19 has affected this vulnerable population on a global scale.

**Methods:**

An online 79-question survey was developed by the International Brain Tumour Alliance, in conjunction with the SNO COVID-19 Task Force. The survey was sent to more than 120 brain tumor charities and not-for-profits worldwide and disseminated to pediatric and adult brain tumor patients and caregivers. Responses were collected from April to May 2020 and subdivided by patient versus caregiver and by geographical region.

**Results:**

In total, 1989 participants completed the survey from 33 countries, including 1459 patients and 530 caregivers. There were no significant differences in COVID-19 testing rates (*P* = .662) or positive cases for brain tumor patients between regions (*P* = .1068). Caregivers were significantly more anxious than patients (*P* ≤ .0001). Patients from the Americas were most likely to have lost their jobs due to the pandemic, practiced self-isolation, and received telehealth services (*P* ≤ .0001). Patients from Europe experienced the most treatment delays (*P* = .0031). Healthcare providers, brain tumor charities, and not-for-profits were ranked as the most trusted sources of information.

**Conclusions:**

As a result of COVID-19, brain tumor patients and caregivers have experienced significant stress and anxiety. We must continue to provide accessible high-quality care, information, and support in the age of COVID-19.

**Key Points**

- Brain tumor patients and caregivers have experienced stress from COVID-19.
- There were no differences in patient testing or positive cases between regions.
- We must continue to provide high-quality care, accessible information, and support to this vulnerable population.

**Importance of the Study**

This is the first international survey completed with the purpose of identifying brain tumor patient and caregiver concerns due to COVID-19. We had a total of approximately 2000 responses consisting of 1500 patients and 500 caregivers from over 30 countries. Results demonstrated that this pandemic has been an extremely stressful time for this population, with caregivers significantly more anxious than patients. There were no significant differences in COVID-19 testing rates or the number of positive cases between regions. Regional differences included that those in the Americas were most likely to have lost their jobs, practiced self-isolation, and received telehealth services. Healthcare providers, brain tumor charities, and not-for-profits were ranked as the most trusted sources of information during the pandemic. This implies a duty for these individuals and organizations to ensure they continue to fulfill patients’ expectations and retain patients’ trust by providing accessible, high-quality care, information, and support.

The novel coronavirus—“COVID-19”—began its unrelenting grip on the world following the first documented case of the disease in the city of Wuhan, Hubei province, China in December 2019.^1^ COVID-19 is also the first documented coronavirus pandemic in history (declared as such on March 11, 2020) and the fifth documented pandemic since the Spanish flu in 1918 which left 500 million people (roughly one-third of the world’s population) infected.^1^ As of the writing of this article in August 2020, and according to the World Health Organization (WHO), COVID-19 has now infected over 17 million people worldwide (confirmed cases) causing more than 650 000 deaths.^1^ At the time this study was conducted, from April to May 2020, COVID-19 went from 1 million confirmed cases on April 2 to over 6 million at the end of May.^1^ Most countries globally went into lockdown after the WHO declared the pandemic on March 11, 2020 and the United States became the country with the highest number of cases.^1^

The COVID-19 pandemic has led to unprecedented stress on the effectiveness and ability of healthcare systems across the globe to function properly. In an effort to free up resources including existing hospital beds, thousands of elective or deemed non-urgent procedures and medical tests have been delayed or canceled. In Canada alone, it is estimated that over 100 000 planned elective procedures (including all oncology and non-oncology) or surgical procedures were canceled or delayed in the first month of the pandemic.^2^ This represents a 60% reduction in the number of planned surgical procedures compared to 2019 data.^2^ A recent global expert response study was conducted to estimate the number of canceled adult elective surgeries worldwide during the 12 weeks of peak disruption due to COVID-19.^3^ The total number of estimated canceled cases was over 28 million, with an overall cancelation rate of 72.3%.^3^ One immediate response to the potential shortage of hospital beds in some countries was to rapidly build, or create from existing buildings, temporary (field) hospitals to increase capacity for treating patients with COVID-19, in order to free up bed space elsewhere and protect patients with other diseases from the virus.^4^

In-person tests and assessments have also either been delayed or changed to telehealth appointments consisting of telephone calls or virtual appointments. Multiple governments around the world have allocated additional resources and funding to telehealth services in an attempt to decrease hospital volumes and virus infection rates.^5^ The current pandemic has significantly threatened every aspect of modern-day life, from countries’ economies to social and family activities. The virus has amplified inequalities and created substantial shifts in human behavior and interpersonal relationships.^6^ It has also strikingly affected the day-to-day management of people with other life-threatening conditions including brain tumors.

Although a number of guidelines for the approach and management of surgical patients have already been suggested and implemented,^7^ oncology patients and specifically neuro-oncology patients are unique because the urgency in their care can rapidly change. While the prioritization of hospital beds and resources to fight COVID-19 is paramount, it is important to continue to provide proper care to the vulnerable brain tumor patient population. To date, the direct effect on brain tumor patients and their caregivers of the drastic healthcare and societal measures implemented during the COVID-19 pandemic is unknown. The purpose of this study is to better understand how COVID-19 and healthcare changes have impacted brain tumor patients and their caregivers on a global scale. By identifying key issues in need of support for this population, we hope to improve the overall care of these patients during the rest of this pandemic and well beyond the recovery from COVID-19.

## Methods

An anonymous online survey in 7 languages (English, French, Italian, German, Spanish, Japanese, and Polish) was developed by the International Brain Tumour Alliance (IBTA) as part of the work of the Society for Neuro-Oncology (SNO) COVID-19 Task Force. Translation support was provided by SNO members and outside translation agencies. The survey questions were first developed by the IBTA and its 13 senior advisors. Following this phase, a wider group, known as the “survey review group,” was sent the draft survey for the final review. The 79-question survey included both categorical and qualitative, open-ended questions. Questions were divided into the following themes: demographics, medical history including brain tumor history, general information and thoughts on COVID-19 and the effect of COVID-19 on treatment delays, telehealth assessments, clinical trials, levels of support from brain tumor charities, and caregiver experiences. Table 1 lists a summary of all 79 questions included in the survey.

**Table 1.** Summary of All Questions

*Demographics*
Who is completing the survey? Patient/patient with help from caregiver/parent or legal guardian of the child
How old are you?
If you live in the United States, what state do you reside in?
If you live outside of the United States, in what country do you live?
How would you describe your gender?
*Medical and brain tumor history*
What type of brain tumor do you have?
When was your brain tumor diagnosed?
Do you have any other medical conditions (“comorbidities”)?
Have you usually, up until now, attended an in-person or virtual (online) brain tumor support group? (2 questions)
Do you have a regular caregiver/not fully independent but do not need a regular caregiver/fully independent?
If you have a caregiver, who is that?
On a scale of 0–100 (where 0 is not feeling anxious at all and 100 is the most anxious), how anxious are you generally feeling about your brain tumor?
What are you doing to cope with these feelings?
Are you currently undergoing active treatment for your brain tumor?
If you are currently undergoing active treatment for your brain tumor, what type of treatment?
*General information about COVID-19*
What do you think your risk of contracting COVID-19 (coronavirus) is?
Do you think that you have been experiencing any COVID-19 (coronavirus) symptoms?
Have you been tested for COVID-19?
Have you been diagnosed with COVID-19?
Are you self-isolating? (with a description of what this means)
On a scale of 0–100 (where 0 is not feeling anxious at all and 100 is the most anxious), how anxious are you about contracting COVID-19?
What are you doing to cope with these feelings?
How do you think you are coping with the challenges of COVID-19?
What is your biggest fear at this time?
How many hours a day do you spend watching TV news, listening to the radio, reading newspaper reports, or going on the Internet for COVID-19 updates?
If you are living in a country with mandatory shutdown and/or self-isolation rules, which of these activities are you able to keep doing?
At this time of the COVID-19 pandemic, please rank from 1 to 6 the way you stay “connected” to the outside world with 1 indicating your favorite communication tool and 6 indicating your least favorite communication tool.
Has COVID-19 affected your medical insurance coverage for treatments or consultations, especially if you are being redirected to a different doctor or clinic that may not be in your local area?
Has the COVID-19 crisis prompted you to have a discussion with your healthcare providers about the management of a life-threatening COVID-19 infection, should it occur?
If you were working prior to the COVID-19 outbreak, do you think your job is now at risk due to the virus pandemic?
Have any members of your immediate family lost their jobs due to the COVID-19 pandemic?
What positive outcomes might there be resulting from alterations in your life caused by the COVID-19 pandemic?
*Effect of COVID-19 on brain tumor information and treatment delays*
How well informed do you feel about how COVID-19 might impact ongoing treatment for your brain tumor?
Who or what is your main source of medical information regarding having a brain tumor during COVID-19? Please rank the following list from 1 to 7 with 1 being your most trusted source and 7 being your least trusted source.
What kind of additional information, if any, would you like to see more of at this time?
Has COVID-19 influenced your or your family’s desire to seek a second opinion at this time?
Has treatment for your brain tumor been delayed or modified in any way by COVID-19?
Which aspects of your brain tumor treatment have been delayed or modified by COVID-19? Select all that apply.
If you are being given the choice to delay any of your treatments, what is influencing your decision?
If you are receiving treatment right now, have you discussed with your treating doctors what the impact of a delay in treatment may mean for you?
If you are currently receiving treatment for your brain tumor, has your treatment been relocated to a different hospital or clinic from where you normally receive it?
Have you personally experienced any shortages of medication specifically for your brain tumor because of COVID-19?
If you answered “yes” to the above question, how are you and your healthcare team dealing with this?
As a result of COVID-19, are there any other changes to your brain tumor treatment that you would like to tell us about?
*Remote/telehealth assessments due to COVID-19*
Has your healthcare provider arranged for remote/virtual/telephone services during COVID-19 to reduce the number of your in-person appointments at your treating center or clinic?
If you answered “yes” to the previous question, is this remote/virtual/telephone care being done in a helpful, efficient, and reassuring manner?
Because of concerns about COVID-19 how willing are you, as a patient, to attend in-person medical appointments at your treating hospital or clinic right now?
If you are attending clinic/hospital appointments right now, have your transportation options been affected because of COVID-19?
If you have answered “yes” to the previous question, how has your attendance at and transportation to your clinic/hospital appointment been affected? Please choose all options which apply.
If you are receiving treatment for your brain tumor right now, how willing are you to skip your next treatments?
If you need to have a scan or other tests for your brain tumor done within the next 6 weeks, how willing are you to go to your treating hospital or clinic to have an MRI or other test?
If you were traveling to another country for your treatment prior to COVID-19, what alternative arrangements have been put in place for you during current travel bans? Please select any options which apply.
How satisfied are you with the care you are getting from your healthcare professionals and their hospitals and clinics at this time?
*Effect of COVID-19 on clinical trials*
If you wanted to enroll in a clinical trial, have you now lost the opportunity to do this because of COVID-19?
If you were considering enrolling in a clinical trial, has COVID-19 affected your decision to enroll (for example, if you are worried about the increasing frequency of visits to the hospital)?
If you were enrolled in a clinical trial, has it changed in any way? For example, have you had to pause your participation in the trial or had trial tests conducted locally rather than where you would normally go?
If you are participating in a clinical trial outside of your own country, how has COVID-19 affected your participation?
*Support for patients and caregivers from brain tumor charities and not-for-profits during COVID-19*
If you are supported by a brain tumor charity or brain tumor not-for-profit in your country, have you noticed any change in their levels of service/information/support during the time of the COVID-19 pandemic?
How satisfied are you with the support you are receiving at this time of COVID-19 from your local, regional, or national brain tumor charity or not-for-profit?
What can your local, regional, or national brain tumor charity/not-for-profit do to support you and your family more through this COVID-19 pandemic?
Final question: is there anything else you would like to add which is not covered by the questions asked above?
*Caregiver specific questions*
How old are you?
How would you describe your gender?
How do you know the brain tumor patient?
On a scale of 0–100 (where 0 is not feeling anxious at all and 100 is the most anxious), how anxious are you generally feeling about your brain tumor?
What are you doing to cope with these feelings?
On a scale of 0–100 (where 0 is not feeling anxious at all and 100 is the most anxious), how anxious are you about contracting COVID-19?
What are you doing to cope with these feelings?
As a caregiver, do you attend an in-person brain tumor support group?
If you do attend an in-person brain tumor support group, do you attend with or without the patient?
As a caregiver, do you attend online support groups?
Are you concerned about being diagnosed with COVID-19?
On a scale of 0–100 (where 0 is not at all and 100 is extreme) how re-traumatized are you about your loved one’s brain tumor as a result of the COVID-19 pandemic?
Has your caring burden increased since the COVID-19 virus started?
What additional burdens and concerns are you facing as a brain tumor caregiver since the COVID-19 virus started? Please select all that apply.
If you are working in paid employment, do you think your job is now at risk due to the virus pandemic?
What, if any, positive outcomes might there be resulting from alterations in your life caused by the COVID-19 pandemic?
Final question: is there anything else you would like to add which is not covered by the questions asked above?

The survey was widely disseminated across the globe to over 120 brain tumor charities and not-for-profits. It was also sent to brain tumor healthcare professionals to send to their pediatric and adult patients and caregivers. The survey collected responses over a 39-day period from April 22 to May 30, 2020, inclusive. Descriptive statistics were performed for all categorical questions. Chi-square tests with pairwise analyses were done with the use of Fisher’s exact test if values were less than 5 to compare patients versus caregivers and between regions. All *P* values less than .05 were considered significant. Questions with less than 10 responses in all categories were not included in statistical analyses. Open-ended, qualitative questions were explored using modified thematic analysis where overarching themes are extracted from the written answers.^8^ This approach uses both open coding (fragments are grouped according to shared ideas) and axial coding (dominant ideas are organized into overarching themes).^5^ All text responses for all questions were independently reviewed by 2 researchers to ensure accurate capture of all relevant overarching themes and ideas. See Supplementary Table 1 for the theme breakdown of qualitative questions.

## Results

In total, 1989 unique surveys were completed from 33 countries, including 48 of 50 states in the United States. These surveys included 1459 patient responses from 1284 adult patients (18 years of age or older) and 175 pediatric patients (younger than 18 years with the parent’s or legal guardian’s assistance) and 530 caregivers. Table 2 lists all countries and the top 3 US states that provided survey responses, including the total number of responses from both patients and caregivers. Of those who answered their current country and state of residence, responses were divided into 3 regions: (1) Americas (North/Central/South America, *n* = 685), (2) Europe (*n* = 257), and (3) Africa/Asia/Oceania (*n* = 278). A total of 530 caregivers completed the caregiver-only section of the survey. The total number of participants including patients and caregivers was 1989. Patient demographics are presented in Table 3. Patient results are summarized and presented in Tables 4–8. Caregiver demographics and results are presented in Table 9.

**Table 2.** List of All Countries With Survey Responses

Country	Number of Responses (Patients and Caregivers)
Anguilla	1
Armenia	1
Australia	142
Belgium	14
Bosnia and Herzegovina	2
Cameroon	2
Canada	163
China	3
Denmark	23
Ecuador	2
France	5
Germany	5
India	5
Indonesia	2
Ireland	26
Italy	43
Japan	185
Kenya	1
Mexico	2
Netherlands	5
New Zealand	10
Norway	3
Poland	28
Singapore	37
South Africa	3
Spain	3
Sweden	21
Switzerland	2
Uganda	2
United Kingdom	173
United States of America: total	805
United States of America: California	101
United States of America: Illinois	95
United States of America: Texas	50
Uruguay	1
Zimbabwe	4
Unanswered	265

**Table 3.** Patient Demographics

Patient Demographics and Medical History for All Patients (*N* = 1459)		
Variable	Responses	*N* (%)
Who is completing the survey?	Adult patient	901 (61.8%)
	Adult patient with help from caregiver	383 (26.3%)
	Pediatric patient	175 (12%)
	Total	1459
Age (years)	0–17	134 (10.6%)
	18–64	968 (76.8%)
	65+	159 (12.6%)
	Total	1261
Gender	Female	701 (55.4%)
	Male	544 (43%)
	Prefer not to say	13 (1%)
	Other	7 (0.6%)
	Total	1265
What country do you live in?	United States of America	566 (46.4%)
	United Kingdom	128 (10.5%)
	Japan	123 (10.1%)
	Canada	117 (9.6%)
	Australia	108 (8.8%)
	Other	179 (14.7%)
	Total	1221
Type of brain tumor	Glioblastoma	288 (22.8%)
	Meningioma	202 (16%)
	Astrocytoma	116 (9.2%)
	Anaplastic astrocytoma	90 (7.1%)
	Anaplastic meningioma	15 (1.2%)
	Anaplastic oligodendroglioma	53 (4.2%)
	Diffuse midline glioma	14 (1.1%)
	Ependymoma	32 (2.5%)
	Medulloblastoma	43 (3.4%)
	Oligodendroglioma	101 (8%)
	Diffuse intrinsic pontine glioma	8 (0.6%)
	Optic nerve glioma	11 (0.9%)
	Unsure	38 (3%)
	Other	255 (20.1%)
	Total	1266
Tumor diagnosis	<3 months ago	44 (3.5%)
	3–5 months ago	68 (5.4%)
	6–12 months ago	143 (11.3%)
	1–5 years ago	506 (40%)
	>5 years ago	246 (19.5%)
	>10 years ago	133 (10.5%)
	>15 years ago	125 (9.9%)
	Total	1265
Do you have any other medical conditions (“comorbidities”)? Select all that apply.	Depression	178 (24.1%)
	High blood pressure	161 (21.8%)
	High cholesterol	123 (16.7%)
	Hypothyroidism	112 (15.2%)
	Arthritis	107 (14.5%)
	Asthma	96 (13%)
	Chronic pain	95 (12.9%)
	Neurological condition	91 (12.3%)
	Osteoporosis/osteopenia	64 (8.7%)
	Diabetes	58 (7.9%)
	Autoimmune disease	47 (6.4%)
	Cardiac disease	33 (4.5%)
	Other	292 (39.6%)
	Total	738
Do you have a caregiver?	Fully independent	686 (54.6%)
	Regular caregiver	377 (30%)
	Not fully independent but no regular caregiver	193 (15.4%)
	Total	1256
If you have a caregiver, who is it?	Partner/spouse	429 (63%)
	Parent(s)	253 (37.2%)
	Children	87 (12.8%)
	Sibling(s)	37 (5.4%)
	Friend	24 (3.5%)
	Other	30 (4.4%)
	Total	681
Are you currently undergoing active treatment for your brain tumor?	Yes	361 (34.9%)
	No	673 (65.1%)
	Total	1034
If you are currently undergoing active treatment for your brain tumor, what type of treatment?	Chemotherapy	207 (33.2%)
	Radiation therapy	80 (12.8%)
	Neurosurgery	57 (9.1%)
	Physiotherapy	64 (10.3%)
	Speech and language therapy	41 (6.6%)
	Palliative care	30 (4.8%)
	Other	144 (23.1%)
	Total	623

**Table 4.** Patient and Caregiver Differences

Question	Responses	*N* (%) or (SD)		*P*
		Patients (*N* = 1459)	Caregivers (*N* = 530)	
On a scale from 0 to 100 (where 0 is not anxious and 100 is most anxious), how anxious are you about the brain tumor?	Mean	50.4 (30.3)	62.9 (30.7)	**<.0001**
	Total	1095	393	
On a scale from 0 to 100 (where 0 is not anxious and 100 is most anxious), how anxious are you about contracting COVID-19?	Mean	48.3 (29.3)	59.3 (30.9)	**<.0001**
	Total	1053	387	
Have you usually, up until now, attended an in-person brain tumor support group?	Yes	313 (24.9%)	84 (24.1%)	.8153
	No	946 (75.1%)	265 (75.9%)	
	Total	1259	349	
Have you usually, up until now, attended an online brain tumor support group?	Yes	258 (20.5%)	94 (24.3%)	.1261
	No	1002 (79.5%)	293 (75.7%)	
	Total	1260	387	
If you are working in paid employment, do you think your job is now at risk due to the pandemic?	Yes	197 (45.6%)	111 (44.9%)	.9309
	No	235 (54.4%)	136 (55.1%)	
	Total	432	247	

Bold meant to highlight all of the significant *P*-values and show which groups they were significant for.

**Table 5.** General COVID-19 Questions by Region

Question	Responses	*N* (%) or (SD)			Overall *P* Value	Significant Pairwise *P* Values
		Americas (North/ Central/South America) (*N* = 685)	Europe (*N* = 257)	Africa/Asia/ Oceania (*N* = 278)		
What do you think your risk of contracting COVID-19 (coronavirus) is?	Same risk as general population	284 (45.9%)	110 (49.3%)	103 (43.5%)	.2387	
	Greater risk than general population	299 (48.3%)	105 (47.1%)	114 (48.1%)		
	Lower risk than general population	36 (5.8%)	8 (3.6%)	20 (8.4%)		
	Total	619	223	237		
Do you think that you have been experiencing any COVID-19 (coronavirus) symptoms?	No	576 (92.9%)	203 (91%)	232 (97.9%)	**.006**	**E vs AAO (.0024)A vs AAO (.0081)**
	Yes	44 (7.1%)	20 (9%)	5 (2.1%)		
	Total	620	223	237		
Have you been tested for COVID-19?	Yes	30 (4.8%)	10 (4.5%)	8 (3.4%)	.662	
	No	590 (95.2%)	213 (95.5%)	227 (96.7%)		
	Total	620	223	235		
Have you been diagnosed with COVID-19?	Yes	6 (1%)	0 (0%)	0 (0%)	.1068	
	No	613 (99%)	222 (100%)	237 (100%)		
	Total	619	222	237		
Are you self-isolating?	Yes	505 (81.6%)	160 (71.7%)	135 (57%)	**<.0001**	**A vs E (.0027)E vs AAO (.0013) A vs AAO (<.0001)**
	No	114 (18.4%)	63 (28.3%)	102 (43%)		
	Total	619	223	237		
How do you think you are coping with the challenges of COVID-19?	Not coping well	25 (4%)	10 (4.3%)	11 (4.6%)	.0125	
	Coping fairly well	213 (34.1%)	85 (36.8%)	114 (47.5%)		
	Coping well	238 (38.1%)	83 (35.9%)	79 (32.9%)		
	Coping very well	149 (23.8%)	53 (22.9%)	36 (15%)		
	Total	625	231	240		
What is your biggest fear at this time?	Contracting COVID-19	185 (45.2%)	64 (38.1%)	98 (59%)	**<.0001**	**A vs E (.0002) E vs AAO (.0001) A vs AAO (.0037)**
	Having my brain tumor surgery delayed	11 (2.7%)	13 (7.7%)	8 (4.8%)		
	Not being able to continue my systemic treatments	67 (16.4%)	48 (28.6%)	18 (10.8%)		
	My caregiver contracting COVID-19	77 (18.8%)	26 (15.5%)	29 (17.5%)		
	Not getting enough support	69 (16.9%)	17 (10.1%)	13 (7.8%)		
	Total	409	168	166		
How many hours a day do you spend on COVID-19 updates?	0 min	30 (4.9%)	5 (2.3%)	3 (1.3%)	**.0009**	**A vs E (.0313) A vs AAO (.0009)**
	<15 min	113 (18.6%)	51 (23.3%)	39 (16.8%)		
	15–60 min	192 (31.6%)	74 (33.8%)	77 (33.2%)		
	1–2 h	129 (21.3%)	55 (25.1%)	75 (32.3%)		
	>2 h	143 (23.6%)	34 (15.5%)	38 (16.4%)		
	Total	607	219	232		
Has the COVID-19 crisis prompted you to have a discussion with your healthcare providers about the management of a life-threatening COVID-19 infection, should it occur?	Yes	95 (18.4%)	32 (17.2%)	63 (30.7%)	**.0005**	**E vs AAO (.0027)A vs AAO (.0005)**
	No	420 (81.6%)	154 (82.8%)	142 (69.3%)		
	Total	515	186	205		
Have any members of your immediate family lost their jobs due to the COVID-19 pandemic?	Yes	170 (32.9%)	36 (18.9%)	33 (16.5%)	**<.0001**	**A vs E (.0004) A vs AAO (<.0001)**
	No	347 (67.1%)	154 (81.1%)	167 (83.5%)		
	Total	517	190	200		

Bold meant to highlight all of the significant *P*-values and show which groups they were significant for.

Abbreviations: A, Americas; E, Europe; AAO, Africa/Asia/Oceania.

**Table 6.** Effect of COVID-19 on Brain Tumors and Treatment Delays by Region

Question	Responses	*N* (%) or (SD)			Overall *P* Value	Significant Pairwise *P* Values
		Americas (North/Central/ South America) (*N* = 685)	Europe (*N* = 257)	Africa/Asia/ Oceania (*N* = 278)		
How well informed do you feel about how COVID-19 might impact ongoing treatment for your brain tumor?	Somewhat informed	108 (19.3%)	45 (21.5%)	61 (28%)	**<.0001**	**A vs E (.004) E vs AAO (.0179) A vs AAO (<.0001)**
	Adequately informed	332 (59.3%)	98 (46.9%)	73 (33.5%)		
	Not well informed	120 (21.4%)	66 (31.6%)	84 (38.5%)		
	Total	560	209	218		
Has treatment for your brain tumor been delayed or modified in any way by COVID-19?	Yes	125 (22.6%)	68 (34.5%)	50 (23.1%)	**.0031**	**A vs E (.0014) E vs AAO (.0145)**
	No	428 (77.4%)	129 (65.5%)	166 (76.9%)		
	Total	553	197	216		
Which aspects of your brain tumor treatment have been delayed or modified by COVID-19? Select all that apply.	Neurosurgery	14 (3.9%)	9 (4.8%)	8 (5.4%)	**.0405**	**A vs E (.0079)**
	Radiation therapy	8 (2.2%)	11 (5.9%)	3 (2%)		
	Chemotherapy	18 (5%)	22 (11.8%)	10 (6.7%)		
	Physiotherapy or speech and language therapy	45 (12.4%)	28 (15%)	22 (14.8%)		
	Follow-up imaging	94 (25.9%)	36 (19.3%)	29 (19.5%)		
	Clinical visits	109 (30%)	46 (24.6%)	40 (26.8%)		
	None	75 (20.7%)	35 (18.7%)	37 (24.8%)		
	Total	363	187	149		
If you are being given the choice to delay any of your treatments, what is influencing your decision?	Travel restrictions	32 (14.3%)	16 (17.8%)	13 (18.1%)	.6916	
	Contracting COVID-19	124 (55.6%)	44 (48.9%)	34 (47.2%)		
	Policy changes	67 (30%)	30 (33.3%)	25 (34.7%)		
	Total	223	90	72		
If you are receiving treatment right now, have you discussed with your treating doctors what the impact of a delay in treatment may mean for you?	Yes	85 (26.2%)	31 (25.4%)	37 (27.4%)	.9329	
	No	240 (73.8%)	91 (74.6%)	98 (72.6%)		
	Total	325	122	135		

Bold meant to highlight all of the significant *P*-values and show which groups they were significant for.

Abbreviations: A, Americas; E, Europe; AAO, Africa/Asia/Oceania.

**Table 7.** Remote and Telehealth Assessments Due to COVID-19 by Region

Question	Responses	*N* (%) or (SD)			Overall *P* Value	Significant Pairwise *P* Values
		Americas (North/Central/ South America) (*N* = 685)	Europe (*N* = 257)	Africa/Asia/Oceania (*N* = 278)		
Has your healthcare provider arranged for remote/virtual/telephone services during COVID-19 to reduce the number of your in-person appointments at your treating center or clinic?	Yes	290 (52.6%)	84 (42.6%)	74 (34.7%)	**<.0001**	**A vs E (.0201) A vs AAO (<.0001)**
	No	261 (47.4%)	113 (57.4%)	139 (65.3%)		
	Total	551	197	213		
Because of concerns about COVID-19 how willing are you, as a patient, to attend in-person medical appointments at your treating hospital or clinic right now?	Unwilling	72 (16.4%)	20 (11.9%)	21 (11.3%)	**.0089**	**A vs E (.0118) A vs AAO (.0281)**
	Willing but very anxious	203 (46.3%)	80 (47.6%)	98 (52.7%)		
	Willing with no concerns	114 (26%)	60 (35.7%)	57 (30.6%)		
	Unsure/uncertain	49 (11.2%)	8 (4.8%)	10 (5.4%)		
	Total	438	168	186		
If you are receiving treatment for your brain tumor right now, how willing are you to skip your next treatments?	Unwilling	118 (64.5%)	55 (68.8%)	69 (74.2%)	.1064	
	Willing but very anxious	33 (18%)	15 (18.8%)	7 (7.5%)		
	Willing with no concerns	8 (4.4%)	5 (6.3%)	8 (8.6%)		
	Unsure/uncertain	24 (13.1%)	5 (6.3%)	9 (9.7%)		
	Total	183	80	93		
If you need to have a scan or other tests for your brain tumor done within the next 6 weeks, how willing are you to go to your treating hospital or clinic to have an MRI or other test?	Unwilling	32 (7.9%)	8 (5%)	13 (7.2%)	.4551	
	Willing but very anxious	222 (54.8%)	87 (54%)	92 (51.1%)		
	Willing with no concerns	129 (31.9%)	62 (38.5%)	66 (36.7%)		
	Unsure/uncertain	22 (5.4%)	4 (2.5%)	9 (5%)		
	Total	405	161	180		
How satisfied are you with the care you are getting from your healthcare professionals and their hospitals and clinics at this time?	Not satisfied	36 (7.2%)	29 (16.4%)	19 (9.4%)	**<.0001**	**A vs E (.0001) A vs AAO (.0006)**
	Satisfied	229 (45.7%)	89 (50.3%)	120 (59.4%)		
	Very satisfied	236 (47.1%)	59 (33.3%)	63 (31.2%)		
	Total	501	177	202		

Bold meant to highlight all of the significant *P*-values and show which groups they were significant for.

Abbreviations: A, Americas; E, Europe; AAO, Africa/Asia/Oceania.

**Table 8.** Effect of COVID-19 on Clinical Trials and Brain Tumor Charities by Region

Question	Responses	*N* (%) or (SD)			Overall *P* Value	Significant Pairwise *P* Values
		Americas (North/Central/ South America) (*N* = 685)	Europe (*N* = 257)	Africa/Asia/ Oceania (*N* = 278)		
If you wanted to enroll in a clinical trial, have you now lost the opportunity to do this because of COVID-19?	Yes	20 (17.1%)	8 (23.5%)	7 (18.9%)	.6967	
	No	97 (82.9%)	26 (76.5%)	30 (81.1%)		
	Total	117	34	37		
If you were considering enrolling in a clinical trial, has COVID-19 affected your decision to enroll (for example, if you are worried about the increasing frequency of visits to the hospital)?	Yes	36 (34.6%)	13 (33.3%)	12 (31.6%)	.9428	
	No	68 (65.4%)	26 (66.7%)	26 (68.4%)		
	Total	104	39	38		
If you were enrolled in a clinical trial, has it changed in any way? For example, have you had to pause your participation in the trial or had trial tests conducted locally rather than where you would normally go?	Yes	8 (16%)	4 (17.4%)	3 (13%)	.9157	
	No	42 (84%)	19 (82.6%)	20 (87%)		
	Total	50	23	23		
How satisfied are you with the support you are receiving at this time of COVID-19 from your local, regional, or national brain tumor charity or not-for-profit?	Not satisfied	24 (13.3%)	14 (16.5%)	12 (17.9%)	.8591	
	Satisfied	79 (43.6%)	36 (42.4%)	30 (44.8%)		
	Very satisfied	78 (43.1%)	35 (41.2%)	25 (37.3%)		
	Total	181	85	67		

**Table 9.** Caregiver Demographics and Results

Caregiver Demographics and Specific Questions (*N* = 530)		
Variable	Responses	*N* (%) or (SD)
Age (years)	18 to 67	480 (90.7%)
	68+	49 (9.3%)
	Total	529
Gender	Male	148 (27.9%)
	Female	378 (71.3%)
	Prefer not to say	1 (0.2%)
	Other	3 (0.6%)
	Total	530
Relationship to patient	Spouse or partner	225 (47.8%)
	Parent	149 (31.6%)
	Sibling	7 (1.5%)
	Another family member	7 (1.5%)
	Friend	3 (0.6%)
	Other	80 (17%)
	Total	471
If you do attend an in-person brain tumor support group, do you attend with the patient?	Yes	64 (55.7%)
	No	51 (44.3%)
	Total	115
Are you concerned about being diagnosed with COVID-19?	Not concerned	54 (13.7%)
	Somewhat concerned	180 (45.7%)
	Very concerned	159 (40.4%)
	I have already been diagnosed with COVID-19	1 (0.3%)
	Total	394
On a scale of 0 to 100 (where 0 is not at all and 100 is extreme), how re-traumatized are you about your loved one’s brain tumor as a result of the COVID- 19 pandemic?	Mean	58.3 (32.8)
	Total	365
Has your caring burden increased since the COVID-19 virus started?	Yes	164 (42.8%)
	No	219 (57.2%)
	Total	383

### Patient Demographics and Medical History

In total, females accounted for 55.4% of responses. From a total of over 30 different brain tumor types reported by patients completing the survey, the 3 most common diagnoses of patients surveyed were glioblastoma (22.8%), meningioma (16%), and astrocytoma (9.2%). The majority of tumor diagnoses of all types reported occurred between 1 and 5 years ago. The most common medical comorbidity in patients was depression (24.1%) followed by high blood pressure (21.8%). A total of 54.6% of patients were described as fully independent. Of those patients with a caregiver, 63% of caregivers were the patient’s partner or spouse. Overall, 34.9% of patients were actively undergoing treatment for their brain tumor at the time of the survey, with chemotherapy accounting for the most common active treatment (33.2%).

### General COVID-19 Questions

In total, 48% of patients believed they were at an increased risk of contracting COVID-19 compared to the general population, independent of both their brain tumor diagnosis and whether or not they were actively undergoing treatment. Significantly more patients in the Americas and Europe compared to Africa, Asia, and Oceania believed they had been experiencing COVID-19 symptoms (*P* = .0081 and .0024, respectively). In total, 4.7% of all patients had been tested for COVID-19 at the time of the survey. There were no significant differences in testing rates for brain tumor patients across regions (*P* = .662). A total of 6 patients of 1459 had been diagnosed with COVID-19, and although all 6 of these patients were from the Americas, there was no significant difference in positive cases between regions (*P* = .1068). Patients in the Americas were self-isolating at higher rates compared to patients from Europe (*P* = .0027), and patients from both the Americas and Europe were self-isolating at higher rates compared to patients in Africa, Asia, and Oceania (*P* ≤ .0001 and .0013, respectively). Overall, 74.1% of patients felt they were coping well or fairly well with the challenges of COVID-19.

Patients in each of the 3 regions—(1) Americas (North/Central/South America, (2) Europe, and (3) Africa/Asia/Oceania—had different fears. Patients in Africa, Asia, and Oceania were significantly more fearful of contracting COVID-19 compared to the Americas and Europe (*P* = .0037 and .0001, respectively). Patients in Europe were most fearful about not being able to continue systemic treatments compared to the Americas and Africa, Asia, and Oceania (*P* = .0002 and .0001, respectively). Patients in the Americas spent more time each day reading or watching COVID-19 updates than patients in either Europe or Africa, Asia, and Oceania (*P* = .0313 and .0009, respectively). Compared to patients in Africa, Asia, and Oceania, patients in the Americas and Europe were less likely to have had a discussion concerning a life-threatening COVID-19 infection with their healthcare providers (*P* = .0005 and .0027, respectively). More patients and their families in the Americas lost their job because of the pandemic compared to both Europe and Africa, Asia, and Oceania (*P* = .0004 and .0001, respectively). For patients and caregivers, 45.4% believe their job is now at risk due to the pandemic.

When answering the qualitative questions, both patients and caregivers mentioned following COVID-19 precautions including using personal protective equipment (PPE), adhering to guidelines, and social distancing was the best way to cope with COVID-19-related anxiety. The majority of patients relied on family and friends to help them cope with the anxiety of their brain tumor, as well as doing hobbies, mindfulness practices, and exercise. The majority of caregivers relied on self-care and mindfulness practices including meditation, positive thinking, and yoga to help them cope.

### Effect of COVID-19 on Brain Tumors and Treatment Delays

Patients in the Americas were the most informed regarding how COVID-19 might impact ongoing treatment for their brain tumor, and patients from Africa, Asia, and Oceania were the least informed (overall *P* ≤ .0001, all pairwise *P* values ≤.05). Europe had significantly more delays and modifications in brain tumor patients’ treatment than both the Americas and Africa, Asia, and Oceania (*P* = .0014 and .0145, respectively). Specifically, compared to the Americas, respondents from Europe indicated more treatment delays (*P* = .079). This included both chemotherapy treatment delays (*P* = .0062) and radiation therapy treatment delays (*P* = .0465). Overall, 68% of patients actively undergoing treatment were unwilling to skip their next treatment. In patients being given the choice to delay treatment, fear of contracting COVID-19 accounts for 52.5% of all influencing factors. Only 26.3% of patients had discussed the impact of treatment delays with their physicians.

In response to the qualitative question of transportation to medical appointments, the majority of patients responded that they drive or are driven by family and avoid using public transportation because of COVID-19.

### Remote and Telehealth Assessments Due to COVID-19

Since COVID-19, remote and telehealth assessments have occurred more in the Americas compared to Europe and Africa, Asia, and Oceania (*P* = .0201 and <.0001, respectively). Patients in the Americas were less willing and more uncertain about attending in-person medical appointments because of concerns about COVID-19 compared to Europe and Africa, Asia, and Oceania (*P* = .0118 and .0281, respectively). In total, 60.9% of patients were either unwilling or willing but very anxious to go to the hospital in the 6 weeks following their completion of the survey for a routinely scheduled test or appointment. At the point in time when patients completed the survey, those in the Americas were more satisfied with the care they were receiving from healthcare professionals compared to Europe and Africa, Asia, and Oceania (*P* = .0001 and .0006, respectively).

In the open-ended question on remote and telehealth appointments, patients reported concern about delayed imaging and clinical appointments as well as overall poorer communication during telehealth assessments.

Doesn’t feel like the doctor understood my problems well through [the] telephone call.

For the first visit in two years, my clinician had to deliver disappointing news of growth – virtually.

### Effect of COVID-19 on Clinical Trials

There were no regional differences between patients in regards to their situation with clinical trials at the time of the COVID-19 survey. In total, 18.6% of patients have lost the ability to enroll in a clinical trial due to COVID-19 at the time of the survey. Of those considering enrolling in a clinical trial, COVID-19 negatively affected 33.7% of patients’ decisions on whether or not they would enroll. For those patients already enrolled in a clinical trial, COVID-19 has led to protocol changes for 15.6% of patients at the time of the survey.

### COVID-19 and the Work of Brain Tumor Charities and Not-For-Profits

Overall, 85% of patients reported that they were satisfied or very satisfied with the support they were receiving from their brain tumor charity or not-for-profit organization during the COVID-19 pandemic.

Patients responded to the qualitative questions about brain tumor charities and not-for-profits noting that many in-person events and meetings scheduled by these organizations had to be canceled because of the pandemic, with some mentioning virtual alternatives replacing these events and others saying no alternative meetings were arranged.

In terms of what these organizations can do to support patients and families during this time, most survey respondents noted that there was nothing more they felt could be done, whereas other responses included suggesting more online support groups or discussions, and more information on topics including COVID-19, brain tumors, and general updates.

The survey revealed that only 23% of patients and caregivers have ever attended an online or in-person brain tumor support group.

Open text answers to the qualitative question on brain tumor charities and not-for-profits included the following examples:

They have stepped up their personal, direct communication with the brain tumor community and came up with creative ways for events to still take place.

They provide useful information on what is available for brain tumour patients in our country. They even offer masks and anti-bacterial wipes.

### General Qualitative Questions

The most common, biggest fear for patients was a delay in their brain tumor-related appointments or treatment.

[I’m worried] that my brain tumor is back and I cannot get the necessary testing done for fear of exposure.

Most patients did not want to see any more information at this time. The majority who did respond to wanting more information wanted more information specifically on how COVID-19 might affect their brain tumor, including associated risk, and treatment implications. Others wanted more information regarding their specific treatment and care plan during the pandemic.

How does [having a] brain tumor affect the likelihood of contracting COVID-19? How does it affect COVID-19 outcome?

When asked about what, if any, positive outcomes might there be from the COVID-19 pandemic, the most frequent response by patients was an improved relationship with family and friends.

We have slowed down and have spent time together as a family in a season that would typically be so busy.

Caregivers also commonly responded that improved family relationships due to self-isolation together were a positive outcome.

When asked if there was anything they would like to add, patients expressed gratitude for the survey or healthcare workers in general and also expressed how anxious or helpless they feel during the pandemic. Caregivers responded by stating the importance of addressing the patient’s needs and quality of life or their feelings of distress as described in this quote: “As a caregiver you want to keep maintaining the patient’s quality of life. Restricting access to family and [the] ability to go out to eat or hike on popular trails or shop in favorite grocery stores or be confined to home on a cold/rainy day – these are things that reduce quality of life.”

### Caregiver Demographics and Differences

Of those caregivers who answered the survey, a total of 90.7% were younger than 68 years and 71.3% were female. The majority of caregivers who responded were the patient’s spouse or partner (47.8%). A total of 21.7% of caregivers have attended any general brain tumor support group, with 55.7% of caregivers attending these support groups with the patients. Overall, 40.4% of caregivers were very concerned about being diagnosed with COVID-19. Caregivers were significantly more anxious than patients concerning brain tumor management and fear of contracting COVID-19 (*P* ≤ .0001).

In total, 42.8% of caregivers felt that their caring burden has significantly increased since the COVID-19 pandemic. The most cited reasons included additional home responsibilities and COVID-19 precautions including the need for PPE, cleaning practices, and social distancing/isolation. In the qualitative responses, access to care was the most cited (23.7%) additional burden or concern that brain tumor caregivers were facing since COVID-19. The burden of worry was highest over concerns that the patient might contract COVID-19 (69% of respondents) and the second highest over fears about taking their loved one to the hospital for treatment and/or monitoring during the pandemic (46.2% of respondents).

Open text (qualitative) responses from the survey highlighted caregivers’ anxiety:

I have no [outside] support from anyone as we are all isolating at home. Also, I am extremely concerned that I will become sick with COVID and there will be no way to protect my [loved one] who has the brain tumor.

I’m not allowed to go to any appointments or treatments [with my loved one who has the brain tumour]. We have to be separated during the darkest days of our lives.

## Discussion

To our knowledge, this is the only international study to date to specifically identify how COVID-19 is affecting brain tumor patients and their caregivers. There was a total of approximately 1500 patient and 500 caregiver responses from 33 countries representing over 30 distinct brain tumor diagnoses. The most prevalent adult and pediatric diagnoses—including glioblastoma, meningioma, and astrocytoma for adults and medulloblastoma, ependymoma, and astrocytoma for children—are representative of the most common adult and pediatric brain tumor diagnoses.^9^ The most common preexisting medical comorbidity in patients was depression, with 24.1% of all brain tumor patients citing this in the medical history section. A recent systematic review and meta-analysis examining the association of depression in brain tumor patients found that overall prevalence of depression or depressive symptoms was 21.7%, closely resembling the responses from this survey.^10^

When answering qualitative questions about how they were managing the stress of a pandemic, the majority of patients said they relied on family and friends to help them cope with the anxiety. Other common coping mechanisms included hobbies, mindfulness practices, and exercise. The majority of caregivers who responded to the qualitative questions on stress relied on self-care and mindfulness practices including meditation, positive thinking, and yoga.

Almost 50% of caregivers felt that their job was now at risk due to the pandemic and that their caring burden since the pandemic began had also increased. According to our survey, more patients and their families from the Americas have lost their jobs because of the pandemic compared to the rest of the world. According to Statistics Canada, in April 2020 alone almost 2 million jobs were lost, with the unemployment rate in March 2020 rapidly rising from 7.8% to 13%.^11^ Similarly in the United States, the unemployment rate reached 14.7% in April 2020 with the loss of over 20 million jobs.^11^ This is the highest rate of unemployment since the Great Depression which occurred from 1929 to the late 1930s.^12^

It was interesting that only approximately 25% of all patients and caregivers prior to the pandemic said that they have ever attended an online or in-person brain tumor charity or not-for-profit support group meeting, especially because this survey was disseminated primarily through these agencies. We would have thought that many more respondents were actively involved in the support group activities that their local or national brain tumor charity or not-for-profit offered. The survey did not ascertain the reason for this low level of in-person or virtual support group attendance. This finding may warrant further investigation. Despite this finding, 85% of patients felt satisfied or very satisfied with the support they were receiving from their brain tumor charity or not-for-profit. Previous research documented a total of 375 adult patients and caregivers attended a brain tumor support group over a 7-year period, with only half of participants attending more than one session for unknown reasons.^13^ In our survey, it appears that low satisfaction with the organization was not a reason for low attendance. When responding to the open-ended questions, patients wanted more specific information on how COVID-19 may specifically affect them and their brain tumor. On a very practical level, a number of survey respondents felt that brain tumor charities and not-for-profits could also provide COVID-19 prevention supplies to them and their families, such as masks and hand sanitizers, as part of their support services at this time.

While almost 50% of patients believed they are at an increased risk of contracting COVID-19 compared to the general population, less than 10% of all patients felt they had been experiencing COVID-19 symptoms. Less than 5% of all patients had been tested for COVID-19 at the time of the survey, and there were only 6 patients who tested positive, all from the Americas. Patients from the Americas and Europe were more likely to believe they had been experiencing COVID-19 symptoms and were also more likely to be self-isolating than those in Africa, Asia, and Oceania.

Overall, brain tumor patients responded that their biggest fear was delayed appointments and treatment due to the pandemic. Between the 3 regions, patients in Africa, Asia, and Oceania were more fearful of contracting COVID-19. Patients in the Americas may be less fearful of contracting COVID-19 because these patients reported spending the most time each day reading or watching COVID-19 updates compared to the other 2 regions. On the other hand, as patients learn more about COVID-19 and spend more time per day on pandemic news this could also increase their fear of contracting the disease.

Patients from Europe were more fearful of not being able to continue systemic treatments due to COVID-19, and this may be reflective of the fact that these patients were less likely to have had a discussion with their healthcare team regarding how a COVID-19 infection may impact their lives. In fact, less than 20% of all patients in the Americas and Europe had a discussion with their healthcare providers about what a diagnosis of COVID-19 might personally mean to them. One explanation for this finding may be that at the time of the survey, COVID-19 had only been present for 2–3 months, and some patients may have 6-month or yearly follow-ups and not yet had the opportunity to discuss this with their healthcare providers.

These findings are even more relevant because, in this survey, patients rated healthcare providers including doctors and nurses as their most trusted source of information. Trust has always been one of the core values at the center of the patient–doctor relationship, and previous research has demonstrated that oncology patients, especially, want an oncologist who is not only knowledgable, but can also accompany them through their illness experience.^14^ Thus, it is extremely important during this time that healthcare providers are available to patients to discuss COVID-19 and its implications for their future treatment.

Regarding treatment delays, patients in the Americas felt the most informed concerning how COVID-19 might impact ongoing treatment for their brain tumor. The results of this question are somewhat at odds with the previous question that less than 20% of all patients had actually discussed what a personal COVID-19 infection might mean with their healthcare providers. This implies that patients from the Americas may be using sources outside of their own healthcare providers to learn about treatment delays due to COVID-19, for example, from the news or other media. Alternatively, it might imply that healthcare providers are talking to patients about treatment delays but failing to provide more specific information regarding COVID-19 to their patients. This is further supported by the fact that only 26.3% of all patients had discussed what the impact of a delay in treatment may mean specifically for them. Overall, it seems that while healthcare providers are discussing general information regarding COVID-19 and treatment delays, there needs to be increased emphasis moving forward on providing risk stratification and treatment plans on a more individualized and patient-specific approach.

More specifically, patients from Europe reported significantly more treatment delays and modifications compared to the rest of the world. Directly comparing Europe to the Americas, patients in Europe reported delays caused by COVID-19 have been to chemotherapy and radiation treatment.

Less than 50% of patients have had remote or telehealth services implemented during COVID-19, with patients in the Americas significantly more likely to have received these services compared to the rest of the world. This may in part be driven by patient preferences. The survey found that those in the Americas are less willing and more uncertain about attending in-person medical appointments due to concerns about catching COVID-19 compared to other regions. This increased reluctance may have led more healthcare providers in these countries to adopt remote or telehealth services such as phone calls or video-conferencing. The survey also found that patients in the Americas were the most satisfied with the care they were currently receiving from healthcare providers, and this may in part be due to increased telehealth services being utilized in these countries. A recent single-institution study from the United States on telemedicine during COVID-19 for neurosurgical patients found that the experience has been overall positive for both patients and physicians, with telemedicine particularly efficient for routine checkups and hardest in cases where a thorough neurological and physical examination was needed.^15^

In our survey, the biggest issues with telehealth assessments were concerns about other forms of healthcare that cannot be done remotely, such as blood work and imaging, as well as some survey respondents citing poor communication taking place during a number of these telehealth assessments. A recurring theme from the open-ended questions was that patients—while appreciative of the care they have been receiving during this time—are wanting more personalized and more specific information regarding their brain tumor or treatment situation and how COVID-19 may directly impact their care moving forward.

In terms of clinical trials, less than 20% of patients at the time of the survey believed they had lost the ability to enroll in a clinical trial due to COVID-19. In reality, medical institutions started canceling clinical trials as early as March, and even at the time of the survey in April–May, most non-COVID-19 clinical trials were shut down.^16^ This likely represents discord between patient knowledge of access to clinical trials compared to the number of clinical trials being offered during the early stages of the pandemic. It was interesting that only one-third of patients felt they would no longer enroll in a clinical trial because of the pandemic. This likely represents patients’ and caregivers’ continued major concerns and anxiety about the brain tumor itself and their willingness to participate in clinical trials if they were offered, regardless of the COVID-19 situation. This represents a crucial dilemma for patients—balancing their fear of COVID-19 with the hope offered by ongoing clinical trials.

Regarding caregivers, the majority of respondents (71.3%) were female and almost half (47.8%) were the patient’s spouse or partner. Caregivers were significantly more anxious than patients about both the patient’s brain tumor and about COVID-19 in general. It is common for caregivers to feel anxious, and there are numerous examples in the literature which have found that caregivers of brain tumor patients often experience depression, anxiety, and decreased quality of life.^17–19^ In responding to the open-ended question regarding coping strategies for COVID-19-induced anxiety, both patients and caregivers felt following COVID-19 precautions best helped them cope, which, due to constantly changing guidelines, procedures, and country-to-country variations in best practice regarding the pandemic, have likely been difficult on respondents. The added stress of COVID-19 and all the uncertainty it has brought is definitely a cause of increased responsibility and anxiety for caregivers. On the other hand, the majority of patients (74.1%) felt that they have been coping well or fairly well with the challenges of COVID-19, and this may in part be due to the excellent ability of their caregivers to adapt and support them during this difficult time. However, this may have come at significant cost to brain tumor caregivers because of the additional burdens they are carrying as a result of the COVID-19 challenges in addition to the challenges they are already facing as caregivers.

The final qualitative questions in the survey asked both patients and caregivers what (if any) additional information they wanted at this time and if there were any positive outcomes from the pandemic thus far. The majority of respondents did not wish to know any additional information at this time. Of those who did, most of the responses focused on wanting to know specific information regarding how COVID-19 may affect them and their brain tumor, including questions as to how their brain tumor may affect the likelihood of contracting COVID-19, and if they get COVID-19 how will that affect their prognosis. While it may be too early to understand specific details of the COVID-19 infection and its relationship to brain tumor treatment and prognosis, it is important to note that these questions are in the minds of brain tumor patients.

Regarding positive outcomes during this time of COVID-19, the overwhelming majority of survey respondents cited the improved relationships and enhanced connections with family and close friends that had arisen from the experience of living through a dangerous pandemic. COVID-19 has made many people reexamine their priorities and has much more appreciation for the simple things in life such as doing good deeds for others, rediscovering and renewing community spirit, and valuing quality time spent with loved ones.

### Limitations

As with all surveys, there may be response bias. The survey consisted of self-selected volunteers and it was not required for survey respondents to complete all questions before submitting the survey. Participants could skip questions and were able to move onto the next question without being blocked from doing this so there is some missing data in the survey results. Additionally, the later questions on average had fewer responses than the earlier questions, possibly due to survey fatigue. Finally, there may have been selection bias because this survey was widely distributed through brain tumor charities and not-for-profits, and the patients and caregivers who completed the survey may not be representative of the entire population. Not all members of the patient and caregiver community have a connection with a brain tumor charity or not-for-profit, and there may be challenges connecting with patients and caregivers from underserved populations, ethnic minorities, and low-income households.

### Future Directions

Overall, COVID-19 has led to unprecedented changes in healthcare delivery and these changes will likely persist and evolve as the pandemic continues. This study highlights the importance of conveying these issues from an institutional as well as a patient-centered approach. Future studies need to address knowledge gaps between healthcare providers and their patients/caregivers and elaborate on refining approaches and protocols for new services including telehealth and virtual appointments. Brain tumor charities and not-for-profits should provide supportive services and objective fact-based information which is reflective of the challenges of the COVID-19 era.

## Conclusions

To our knowledge, this is the only international survey completed to date with the purpose of identifying brain tumor patient and caregiver concerns due to the COVID-19 pandemic. Our survey highlights important key issues.

First, the pandemic has been an extremely stressful time for the brain tumor patient and caregiver population. In addition to dealing with general societal issues such as job security and COVID-19 guidelines, brain tumor patients and caregivers have also had to deal with uncertainty about their medical care and the adoption of telehealth services. A brain tumor diagnosis brings with it a great deal of uncertainty in any event. But the arrival of COVID-19 has substantially amplified this. While the pandemic has been stressful for both patients and caregivers, caregivers were significantly more anxious than patients. So, providing support and reassurance not only to brain tumor patients but also to caregivers is crucial during this doubly difficult time.

Second, regional differences in the approach to and care of brain tumor patients during COVID-19, and the far-reaching societal effects of COVID-19, were evident in the survey. Patients in the Americas were more likely to have lost their jobs, believed they have experienced COVID-19 symptoms, practiced self-isolation, and received telehealth services. Patients from Europe reported more treatment delays. Those from Africa, Asia, and Oceania were most fearful about contracting COVID-19.

Third, patients worldwide wanted more specific information from their healthcare providers and brain tumor charities and not-for-profits about the interplay between COVID-19 and their brain tumor diagnosis. Moving forward, there needs to be an increased emphasis on research and communication concerning issues such as prognostication and risk stratification for brain tumor patients with regard to COVID-19. The brain tumor community should strive to provide meaningful COVID-19 information and support to patients and caregivers as further research adds to our knowledge of this virus and its effects.

Finally, patients and caregivers ranked healthcare providers and brain tumor charities and not-for-profits as their most trusted sources of information during the challenging times of a coronavirus pandemic. This implies a duty for healthcare professionals and brain tumor charities and not-for-profits to ensure that they continue to fulfill patients’ expectations and retain patients’ confidence and trust by providing accessible, high-quality care, information, and support in the age of COVID-19.

## Supplementary Material

vdaa104_suppl_Supplementary_Table_1

## International Brain Tumour Alliance (IBTA) Senior Advisors

The list of IBTA Senior Advisors includes as follows: Jean Arzbaecher, Carol Kruchko, Mary Ellen Maher, Chris Tse, Rosemary Cashman, Maureen Daniels, Christine Mungoshi, Sharon Lamb, Anita Granero, Mary Lovely, Jenifer Baker, and Sally Payne.

## Notes

**Authorship statement.** Design of the survey and question selection: K.O., S.F., and IBTA Senior Advisors. Dissemination of survey: K.O., S.F., and IBTA Senior Advisors. Collection of all survey responses: K.O. and S.F. Analysis of all survey responses: M.R.V., T.C., and G.Z. Manuscript preparation: M.R.V., T.C., K.O., S.F., and G.Z. All authors read, edited, and approved the final manuscript.
