# Supplementary material for: Brain tumors and COVID-19: the patient and caregiver experience
Source: Neurooncol Adv. 2020 Aug 23;2(1):vdaa104. doi: 10.1093/noajnl/vdaa104 (PMC7499687; doi:10.1093/noajnl/vdaa104)
Supplement: vdaa104_suppl_Supplementary_Table_1 [file vdaa104_suppl_supplementary_table_1.docx]

**Supplementary Table 1**

| **Supplementary Table 1: Qualitative Question Code and Theme Summary** | | |
| --- | --- | --- |
| **Patient survey questions** | | |
| **Question 19: What are you doing to cope with these feelings? (Brain tumour anxiety)** | | **Respondents** |
|  |  | 848 |
| **Hobbies** | **Medication** | **Exercise** |
| 132 | 56 | 136 |
| **Mindfulness/Positive Thinking** | **Family/Friends** | **Avoidance** |
| 178 | 182 | 67 |
| **Religion** | **Sleep/Rest** | **Maintain Routine** |
| 73 | 25 | 52 |
| **Nothing** | **External Support Groups, Therapy etc** | **Work/School** |
| 84 | 94 | 34 |
| **Nutriton** | **Stay informed on health issues** | **Other** |
| 9 | 62 | 6 |
| **Question 21: What are you doing to cope with these feelings? (COVID-19 anxiety)** | | **Respondents** |
|  |  | 794 |
| **Hobbies** | **Religion** | **Covid Precautions** |
| 49 | 30 | 385 |
| **Nothing** | **Nutrition** | **Mindfulness** |
| 96 | 10 | 78 |
| **Exercise** | **Family/Friends** | **Professional Support/Therapy** |
| 51 | 65 | 23 |
| **Emotional (crying,panic)** | **Other** | **Info on Research** |
| 6 | 11 | 26 |
| **Following HCW Advice** | **Avoidance** | **Normal Routine** |
| 6 | 58 | 18 |
| **Medication** |  |  |
| 11 |  |  |
| **Question 23: What is your biggest fear at this time?** | | **Respondents** |
|  |  | 229 |
| **Brain tumour/cancer** | **Delayed Appointmt/treatment** | **Family/Friends** |
| 61 | 71 | 47 |
| **Contracting COVID (family, themselves)** | **The Future** | **Mental Health** |
| 42 | 12 | 10 |
| **Economy/Career** | **Other** |  |
| 19 | 14 |  |
| **Question 29: What kind of additional information, if any, would you like to see more of at this time?** | | **Respondents** |
|  |  | 372 |
| **None** | **Info on Care Plan** | **Tumour/Treatmt Info** |
| 113 | 53 | 52 |
| **Support Group** | **Connection with Care Team** | **COVID research (and brain tumours)** |
| 10 | 29 | 83 |
| **Evolution of Pandemic** | **Other** | **More Detailed/Recent Info** |
| 8 | 18 | 10 |
| **Online Articles & Info Session** | **Children** | **COVID Precautions** |
| 5 | 5 | 5 |
| **Anxiety** |  |  |
| 2 |  |  |
| **Question 37: Is this remote/virtual/telephone care being done in a helpful, efficient and reassuring manner? If No, please explain why:** | | **Respondents** |
|  |  | 49 |
| **Imaging/Tests** | **In-person assessment** | **Technology** |
| 13 | 7 | 4 |
| **Inefficent** | **Other** | **Poor Communication** |
| 7 | 8 | 10 |
| **Change in Care** |  |  |
| 4 |  |  |
| **Question 45: How has your attendance at and transportation to your clinic/hospital appointment been affected? Other please specify:** | | **Respondents** |
|  |  | 88 |
| **Family** | **Driving** | **No air travel** |
| 26 | 43 | 7 |
| **Less visits** | **Ambulance** | **(Avoid/Less) Public Transit** |
| 3 | 2 | 14 |
| **Telehealth/ No Appointmt** | **Other** |  |
| 3 | 6 |  |
| **Question 51: As a result of COVID-19, are there any other changes to your brain tumour treatment that you would like to tell us about?** | | **Respondents** |
|  |  | 54 |
| **Assistance/Access to care** | **Appointmt Delays** | **Abandonment/Distress** |
| 7 | 26 | 4 |
| **Positive Change** | **Need for Support** | **Transportation** |
| 2 | 5 | 2 |
| **Insurance** | **Other** |  |
| 3 | 5 |  |
| **Question 58: If you are supported by a brain tumour charity or brain tumour not-for-profit in your country, have you noticed any change in their levels of service/information/support during the time of the COVID-19** | | **Respondents** |
|  |  | 78 |
| **Cancelled meetings/events** | **Less funding/financial** | **More support** |
| 35 | 5 | 14 |
| **Virtual Meetings/adaptations** | **Pandemic info** | **Less info/support** |
| 13 | 6 | 5 |
| **Other** |  |  |
| 3 |  |  |
| **Question 60: What can your local, regional or national brain tumour charity/not-for-profit do to support you and your family more through this COVID-19 pandemic?** | | **Respondents** |
|  |  | 239 |
| **Advocacy** | **COVID-19 updates** | **Support Group/Emotional Support** |
| 8 | 22 | 32 |
| **Financial** | **Provide Information** | **Satisfied** |
| 11 | 36 | 22 |
| **None/NA** | **Other** | **Online Discussions/Videos** |
| 63 | 16 | 33 |
| **Transportation Support** | **Provide PPE and Sanitizing Materials** |  |
| 4 | 12 |  |
| **Question 61: COVID-19 has created a lot of uncertainty and turbulence for everyone including, of course, all of those in the brain tumour community, no matter what their role is. But what positive outcomes might there be resulting from alterations in your life caused by the COVID-19 pandemic? In your relationship with others / In healthcare delivery** | | **Respondents** |
|  |  | 344 |
| **None** | **Virtual Care/Technology** | **Family/Friends** |
| 90 | 56 | 90 |
| **Gratitude/Reflection** | **Health Safety Measures** | **Other** |
| 45 | 37 | 23 |
| **Less Stress, Slower Pace** | **Support groups** | **Education/Research** |
| 20 | 2 | 4 |
| **Question 62: Is there anything else you would like to add which is not covered by the questions asked above?** | | **Respondents** |
|  |  | 248 |
| **No** | **Fear of COVID** | **Gratitude/Thankfulness** |
| 116 | 4 | 43 |
| **Helplessness/Anxiety** | **Covid Policy** | **Survivorship/Acceptance** |
| 39 | 9 | 9 |
| **Supporting Others/Community** | **Survey** | **Other** |
| 11 | 8 | 7 |
| **Personal Support** | **Info on Health Topics** |  |
| 4 | 19 |  |
| **Caregiver Survey Questions** | | |
| **Question 67: What are you doing to cope with these feelings? (Brain tumour anxiety)** | | **Respondents** |
|  |  | 266 |
| **Exercise** | **Family/Friends** | **Religion** |
| 34 | 42 | 28 |
| **Self Care/Mindfulness (meditation, yoga)** | **Work** | **Therapy/Support** |
| 62 | 9 | 37 |
| **Avoidance** | **Nothing** | **Normal routine** |
| 25 | 26 | 10 |
| **Support to Patient (attend appointments, care)** | **Research** | **COVID precautions** |
| 29 | 21 | 9 |
| **Hobbies** | **Others** | **Medication** |
| 22 | 9 | 3 |
| **Question 69: What are you doing to cope with these feelings? (COVID-19 anxiety)** | | **Respondents** |
|  |  | 254 |
| **COVID Precautions** | **Informed/Education** | **Nothing** |
| 137 | 15 | 22 |
| **Hobbies** | **Family/Friends** | **Exercise** |
| 19 | 22 | 22 |
| **Nutrition** | **Religion** | **Other** |
| 3 | 11 | 7 |
| **Support/Therapy** | **Avoidance** | **Mindfulness** |
| 10 | 19 | 22 |
| **Helplessness/Anxiety** |  |  |
| 12 |  |  |
| **Question 75: Has your caring burden increased since the COVID-19 virus started?** | | **Respondents** |
|  |  | 156 |
| **Stress/Burden** | **Anxiety** | **Less Family/Friend Interactions** |
| 25 | 24 | 11 |
| **COVID Precautions** | **No Visiting** | **Kids at Home/Homeschool** |
| 47 | 9 | 14 |
| **No in Person Support** | **More care/Care Coord** | **Other** |
| 11 | 9 | 9 |
| **Less Income** | **Working at Home** | **Household Responsibility** |
| 5 | 11 | 46 |
| **Question 76: What additional burdens and concerns are you facing as a brain tumour caregiver since the COVID-19 virus started? Other please specify:** | | **Respondents** |
|  |  | 38 |
| **Finances** | **Contracting COVID** | **Treatment plan/Access to care** |
| 4 | 4 | 9 |
| **Work** | **Family Care** | **Other** |
| 4 | 4 | 6 |
| **Social Distance/Isolation** | **Visitors** |  |
| 7 | 4 |  |
| **Question 78: COVID-19 has created a lot of uncertainty and turbulence for everyone including, of course, all of those in the brain tumour community, no matter what their role is. But what, if any, positive outcomes might there be resulting from alterations in your life caused by the COVID-19 pandemic? in relationships with others / in healthcare delivery** | | **Respondents** |
|  |  | 171 |
| **None** | **Telehealth/Online care** | **Family/Social Relationships Improved** |
| 17 | 18 | 69 |
| **Gratitude** | **More Free Time** | **Money/Finances** |
| 12 | 18 | 3 |
| **Other** | **Better Health** |  |
| 16 | 10 |  |
| **Question 79: Is there anything else you would like to add which is not covered by the questions asked above?** | | **Respondents** |
|  |  | 87 |
| **Grief** | **Patient Success** | **Economy** |
| 3 | 2 | 4 |
| **No** | **Distress** | **Visitor Policy** |
| 46 | 14 | 2 |
| **Other** | **Support Groups** | **Anxiety** |
| 6 | 3 | 9 |
| **Patient Needs** | **Satisfied with Care** |  |
| 11 | 2 |  |
